# Nephrectomies in Saudi Arabia: A Comprehensive Analysis of Outcomes from a High-Volume Minimally Invasive Surgery Center

**DOI:** 10.15586/jkcvhl.v11i3.332

**Published:** 2024-07-18

**Authors:** Ahmed Alasker, Turki Rashed Alnafisah, Areez Shafqat, Belal Nedal Sabbah, Marwan Alaswad, Mohammad Alghafees, Abdullah Alhaider, Abdulrahman Alsayyari, Naif Althonayan, Mohammed Al-Otaibi, Salman Bin Ofisan, Mohammed Ghazi Alharbi, Bader Alsaikhan, Abdullah Al-Khayal

**Affiliations:** 1 College of Medicine, King Saud bin Abdulaziz University for Health Sciences, Riyadh, Kingdom of Saudi Arabia;; 2 Department of Urology, King Abdulaziz Medical City, Riyadh, Kingdom of Saudi Arabia;; 3 Department of Medicine, King Abdullah International Medical Research Center, Riyadh, Kingdom of Saudi Arabia;; 4 College of Medicine, Alfaisal University, Riyadh, Kingdom of Saudi Arabia;; 5 College of Medicine, Prince Sattam Bin Abdulaziz University, Al-Kharj, Kingdom of Saudi Arabia

**Keywords:** Nephrectomy, Minimally Invasive Surgery, Nephrectomy Outcomes, Saudi Arabia

## Abstract

Literature reporting on the outcomes of the different procedures of nephrectomies (open vs laparoscopic vs robotic) in Saudi Arabia remains limited. Compare surgical and oncological outcomes between open and minimally invasive nephrectomies. A retrospective cohort study. The present study included all adult patients who underwent nephrectomies between January 1, 2015 and January 31, 2023. We collected demographic, preoperative, intraoperative, and postoperative data on 408 adult cancer patients who underwent nephrectomies at our center between January 2015 and January 2023. Statistical differences were calculated between procedure types. Overall survival was calculated using Kaplan–Meier curves with log-rank tests. P<0.05 was considered statistically significant. Measures of operative success (intraoperative blood loss, intraoperative and postoperative complications, and hospital stay) and oncological outcomes (local recurrence, metastatic progression, and chemotherapy use) between different procedure and nephrectomy types for cancer patients. A total of 408 cancer patients underwent nephrectomies. In cancer patients, open nephrectomy was associated with significantly higher intraoperative blood loss (p<0.001), incidence of blood transfusions (p<0.001), hospital stay (p<0.001), intraoperative complications (p=0.027 and p=0.001, respectively), local recurrence (p<0.001), metastatic progression (p=0.001), and chemotherapy (p=0.001) than minimally invasive surgery, but survival differences across procedure types were not statistically significant (log-rank p-value = 0.054). Regarding nephrectomy type, significant differences were observed in tumor size (p < 0.001), initial procedure type (p<0.001), operation time (p<0.001), blood transfusion (p=0.033), length of hospital stay (p=0.004), intraoperative complications (p=0.020), postoperative complications (p=0.025), Clavien classification (p=0.003), mortality (p=0.022), metastatic progression (p<0.001), and chemotherapy use (p=0.001) between simple/total nephrectomy, radical nephrectomy (RN), partial nephrectomy (PN), and nephroureterectomy. Survival differences between the four nephrectomy types were statistically significant (log-rank p value = 0.001). Minimally invasive nephrectomies reduce inpatient morbidity while conferring equivalent oncological and surgical outcomes.

## Introduction

Nephrectomy, a surgical procedure involving the removal of one or both kidneys, is commonly performed to treat various kidney diseases, such as renal cancer and end-stage kidney disease (ESKD). The main types of nephrectomies include radical and partial nephrectomy, with the latter considered nephron-sparing (1). The American Urological Association guidelines recommend partial nephrectomy as a first-line treatment for tumors between 4 and 7 cm in size—clinically classified as stage T1a or T1b—or for patients with absolute indications such as bilateral renal masses or a solitary kidney (2). Regardless of the type, nephrectomy has witnessed a significant shift toward minimally invasive surgery (MIS)—comprising laparoscopic and robotic approaches—in recent years due to its numerous advantages over traditional open surgery such as reduced perioperative blood loss and analgesia use, shorter hospital stay, quicker return to full function, and equivalent short- and long-term oncological outcomes (3–8). Both radical and partial nephrectomies can be performed openly or using minimally invasive laparoscopic and robotic approaches (9).

Comparisons of open and minimally invasive nephrectomy encompassing surgical and oncological outcomes in Saudi Arabia are limited. With the projected 33% increase in kidney cancer cases and the growing number of ESKD patients in Saudi Arabia, the burden for nephrectomies is naturally expected to rise (10, 11). Alhaidari et al. compared open nephrectomy (n = 42) to robotic nephrectomy (n = 28) in a cohort of 70 renal cancer patients, revealing significantly lower estimated blood loss and shorter hospital stay in the latter (12). Another regional study by Seyam et al. in 2019 retrospectively examined 101 patients undergoing robotic partial nephrectomy and reported favorable outcomes in terms of blood loss, warm ischemia time, operative time, and complication rate (13). They found that 73% of patients achieved a trifecta, which is the most common criterion used for assessing short-term nephrectomy outcome (13). However, this study lacked a comparison group, was limited by small sample size, and lacked an assessment of long-term surgical and oncological outcomes. A subsequent study by our group was the first regional study to compare partial nephrectomy outcomes based on procedure type (open, laparoscopic, and robotic) (14). However, experience with robot-assisted partial nephrectomy was limited in Saudi Arabia at the time and hence outcomes were not comparable to high-volume centers in the West (14).

To expand upon these findings, we present a detailed comparison of the surgical and oncological outcomes between open and minimally invasive nephrectomies at our center which boasts a high urology operating room volume of 4,000 procedures annually, encompassing all variations of nephrectomies.

## Patients and Methods

### Patient population

The present study included all adult patients who underwent nephrectomies between January 1, 2015, and January 31, 2023. Patients aged under 18 years and those who had undergone nephrectomies at another center but were following up at our center were excluded from the study. Demographic, preoperative, intraoperative, and postoperative variables were collected from the BESTCare system (ezCareTech, South Korea). Figures were created by Microsoft Excel 2019 (Microsoft Corporation, WA, USA) and statistical analysis was performed using the Statistical Package for the Social Sciences (SPSS) version 23.0 (IBM Corporation, NY, USA).

### Statistical analysis

Categorical variables were presented as frequencies and percentages, while numerical variables were expressed as median and interquartile ranges (IQRs). Statistical differences between procedure types were assessed using Pearson’s Chi-squared test or Fisher’s exact test. The Wilcoxon rank sum test was used to evaluate differences between nephrectomy types (i.e., radical or partial) and the Kruskal–Wallis rank sum test was employed to assess differences between procedure types (open, laparoscopic, or robotic). Analysis of variance (ANOVA) was applied to explore the interaction between time (preoperative, 3–6 months, and 6–12 months) and procedure type on numerous laboratory parameters. Time was considered a within-subject effect, while procedure type was a between-subjects variable. Kaplan–Meier plots were used to depict survival curves, and differences in survival were assessed using a log-rank test. A p-value of < 0.05 indicated statistical significance.

### Ethical considerations

The study was approved by the Institutional Review Board of King Abdullah International Medical Research Center, Ministry of National Guard-Health Affairs, Riyadh, Kingdom of Saudi Arabia (approval number RC18/163/R). Patient confidentiality was ensured.

## Results

Data of a total of 408 cancer patients undergoing nephrectomies were included. The median age of patients was 59.0 years (IQR 49.5.0– 68.0), with a median BMI of 30.3 kg/m^2^ (IQR 25.7–34.0). Most of our patients were male (63.2%) and Saudis (93.1%). More details about the demographic characteristics are provided in Table 1.

**Table 1:** Demographic characteristics of renal cancer patients who underwent nephrectomies.

Parameter	Category	Cancer cohort	
		Description (n=408)	Missing
Age	Years	59.0 (49.5, 68.0)	1 (0.2%)
Height	m	1.7 (1.6, 1.7)	0 (0%)
Weight	kg	80.0 (69.0, 91.7)	0 (0%)
BMI	kg/m^2^	30.3 (25.7, 34.0)	0 (0%)
Gender	Male	258 (63.2%)	0 (0%)
	Female	150 (36.8%)	
Nationality	Saudi	379 (93.1%)	1 (0.2%)
	Non-Saudi	28 (6.9%)	
Past Medical History (Renal)	None	301 (74.3%)	3 (0.7%)
	Pyeloplasty	0 (0.0%)	
	Past renal surgery	7 (1.7%)	
	Other	97 (24.0%)	

*Categorical variables are described as frequencies (percentages), and numerical data are described as median (IQR).

### Operative characteristics and outcomes of patients with cancer by procedure type

**Figure 1:**
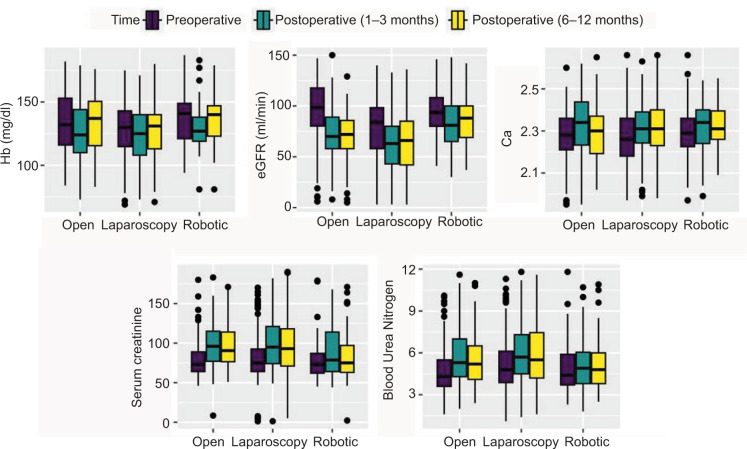
Box plots of the concentration of laboratory parameters across different time points and procedure types among patients with cancer (n = 408).

**Figure 2:**
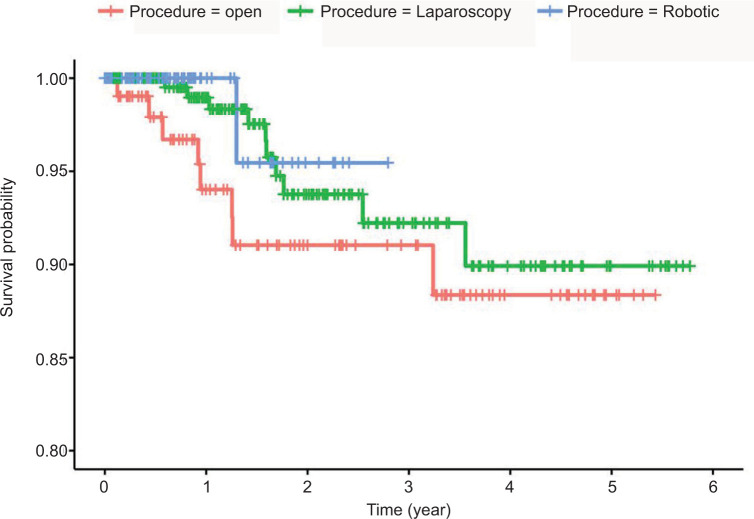
A Kaplan–Meier plot depicting survival curves across different procedure types among patients with cancer (n = 408).

Robotic surgeries had significantly higher rates of partial nephrectomy (90.7%) compared to laparoscopic (16.6%) and open surgeries (38.6%, p < 0.001) (Table 2). In addition, open nephrectomies were associated with significantly higher blood loss (p <0.001), rates of blood transfusions (p < 0.001), intraoperative complications (p = 0.001), and postoperative complications (p = 0.028) than MIS approaches. We used the Clavien grading system for classifying intraoperative adverse events and found a significant difference between open, laparoscopic, and robotic nephrectomies (p = 0.016). Open procedures were significantly associated with higher rates of local recurrence (p < 0.001), metastatic progression (p = 0.001), and use of chemotherapy (p = 0.001) (Table 2). Analysis of laboratory parameters revealed a significant interaction between time and procedure type for changes in blood urea nitrogen (p = 0.016), while other laboratory indices did not show significant interactions (Figure 1). Death occurred among 4.7% of patients with cancer, with rates of 11.4% in open nephrectomy, 4.1% in laparoscopic nephrectomy group, and 1.0% in the robotic nephrectomy group. However, the difference in survival was not statistically significant across different procedure types (log-rank p value = 0.054, Figure 2).

**Table 2:** Cancer-related and operative characteristics and outcomes of patients with cancer by the procedure type.

Parameter	Category	Overall, N = 408	Procedure type			p-value	Missing
			Open, N = 70	Laparoscopy, N = 241	Robotic, N = 97		
Tumor Size	cm	4.5 (3.0, 7.0)	7.0 (4.0, 12.4)	5.0 (3.5, 7.6)	3.0 (2.0, 4.0)	<0.001	43 (11%)
Stage T	TX	0 (0.0%)	0 (0.0%)	0 (0.0%)	0 (0.0%)	<0.001	60 (15%)
	T0	0 (0.0%)	0 (0.0%)	0 (0.0%)	0 (0.0%)		
	T1	171 (49.1%)	17 (28.8%)	82 (39.8%)	72 (86.7%)		
	T2	20 (5.7%)	8 (13.6%)	12 (5.8%)	0 (0.0%)		
	T3	157 (45.1%)	34 (57.6%)	112 (54.4%)	11 (13.3%)		
Stage N	NX	305 (88.4%)	41 (67.2%)	182 (90.5%)	82 (98.8%)	<0.001	63 (15%)
	N0	25 (7.2%)	12 (19.7%)	13 (6.5%)	0 (0.0%)		
	N1	13 (3.8%)	7 (11.5%)	5 (2.5%)	1 (1.2%)		
	N2	1 (0.3%)	0 (0.0%)	1 (0.5%)	0 (0.0%)		
	N3	1 (0.3%)	1 (1.6%)	0 (0.0%)	0 (0.0%)		
Stage M	MX	342 (97.2%)	55 (87.3%)	204 (99.0%)	83 (100.0%)	<0.001	56 (14%)
	M0	2 (0.6%)	2 (3.2%)	0 (0.0%)	0 (0.0%)		
	M1	8 (2.3%)	6 (9.5%)	2 (1.0%)	0 (0.0%)		
Nephrectomy Type	Radical	231 (56.6%)	38 (54.3%)	187 (77.6%)	6 (6.2%)	<0.001	0 (0%)
	Partial	155 (38.0%)	27 (38.6%)	40 (16.6%)	88 (90.7%)		
	Simple/total	3 (0.7%)	2 (2.9%)	1 (0.4%)	0 (0.0%)		
	Nephroureterectomy	19 (4.7%)	3 (4.3%)	13 (5.4%)	3 (3.1%)		
Side	Right	199 (49.1%)	33 (47.8%)	115 (48.1%)	51 (52.6%)	0.360	3 (0.7%)
	Left	196 (48.4%)	33 (47.8%)	117 (49.0%)	46 (47.4%)		
	Both	10 (2.5%)	3 (4.3%)	7 (2.9%)	0 (0.0%)		
Converted to Open Surgery	No	343 (95.5%)	26 (100.0%)	225 (93.8%)	92 (98.9%)	0.078	49 (12%)
	Yes	16 (4.5%)	0 (0.0%)	15 (6.3%)	1 (1.1%)		
Reasons of Conversion*	Bleeding	6 (40.0%)	0 (NA%)	6 (42.9%)	0 (0.0%)	0.600	1 (6.3%)
	Difficult dissection	3 (20.0%)	0 (NA%)	3 (21.4%)	0 (0.0%)		
	Failure to progress	2 (13.3%)	0 (NA%)	2 (14.3%)	0 (0.0%)		
	Obesity	1 (6.7%)	0 (NA%)	1 (7.1%)	0 (0.0%)		
	Other	3 (20.0%)	0 (NA%)	2 (14.3%)	1 (100.0%)		
Operation Time (min)	Minutes	202.0 (161.0, 258.0)	228.0 (178.0, 295.0)	197.0 (160.0, 258.0)	197.0 (158.5, 247.8)	0.234	136 (33%)
Est. blood loss (mL)	mL	200.0 (150.0, 500.0)	500.0 (300.0, 1,000.0)	200.0 (100.0, 400.0)	200.0 (100.0, 245.0)	<0.001	128 (31%)
Blood Transfusion	Yes	25 (6.3%)	12 (17.1%)	12 (5.1%)	1 (1.1%)	<0.001	9 (2.2%)
Hospital Stay (days)	Days	5.0 (3.0, 8.0)	8.0 (6.0, 13.0)	5.0 (3.0, 8.0)	4.0 (3.0, 5.0)	<0.001	4 (1.0%)
Intraoperative Complications	Yes	17 (4.2%)	8 (11.4%)	9 (3.7%)	0 (0.0%)	0.001	0 (0%)
Postoperative Complications	Yes	34 (8.3%)	11 (15.7%)	14 (5.8%)	9 (9.3%)	0.028	0 (0%)
Clavien Classification	No complications	307 (82.1%)	41 (68.3%)	196 (84.8%)	70 (84.3%)	0.016	34 (8.3%)
	1	15 (4.0%)	2 (3.3%)	8 (3.5%)	5 (6.0%)		
	2	10 (2.7%)	4 (6.7%)	5 (2.2%)	1 (1.2%)		
	3	14 (3.7%)	3 (5.0%)	6 (2.6%)	5 (6.0%)		
	4	12 (3.2%)	6 (10.0%)	6 (2.6%)	0 (0.0%)		
	None	16 (4.3%)	4 (6.7%)	10 (4.3%)	2 (2.4%)		
Local Recurrence	Yes	12 (3.1%)	7 (11.1%)	5 (2.1%)	0 (0.0%)	<0.001	20 (4.9%)
Metastatic Progression	Yes	44 (11.3%)	16 (24.2%)	21 (9.1%)	7 (7.6%)	0.001	20 (4.9%)
Chemotherapy	Yes	26 (6.7%)	12 (18.2%)	11 (4.7%)	3 (3.3%)	0.001	18 (4.4%)

*Descriptive data are based on 16 records of patients who had their surgeries converted to open procedures.

### Operative characteristics and outcomes of patients with cancer by nephrectomy type

**Figure 3:**
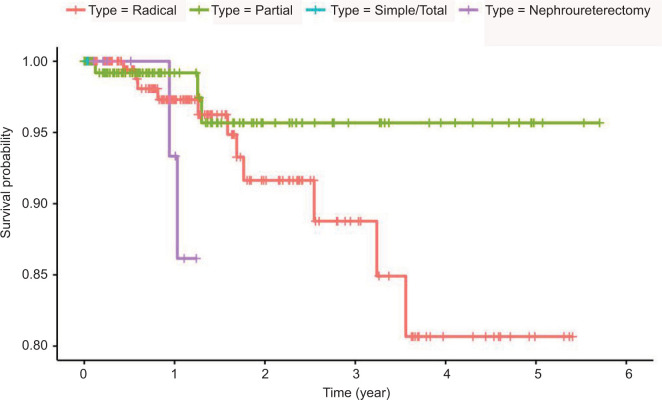
A Kaplan–Meier plot depicting survival curves across types of nephrectomies among patients with cancer (n = 147).

Significant differences were also observed between different nephrectomy types in terms of tumor size (p < 0.001), initial procedure type (p < 0.001), operation time (p < 0.001), blood transfusion (p = 0.033), length of hospital stay (p = 0.004), intraoperative complications (p = 0.020), postoperative complications (p = 0.025), and Clavien classification (p = 0.003), as well as the rates of death (p = 0.022), metastatic progression (p < 0.001), and chemotherapy use (p = 0.001, Table 3). Differences in patient survival between the four types of nephrectomies were statistically significant (log rank p value < 0.001, Figure 3).

**Table 3:** Cancer-related and operative characteristics and outcomes of patients with cancer by the type of nephrectomy.

Parameter	Category	Overall, N = 408	Nephrectomy type				p-value	Missing
			Radical, N = 231	Partial, N = 155	Simple/Total, N = 3	Nephroureterectomy, N = 19		
Tumor Size (cm)	cm	4.5 (3.0, 7.0)	6.1 (4.5, 9.0)	3.0 (2.0, 4.0)	12.0 (7.5, 13.0)	6.0 (4.0, 9.3)	<0.001	43 (11%)
Stage T	TX	0 (0.0%)	0 (0.0%)	0 (0.0%)	0 (0.0%)	0 (0.0%)	<0.001	60 (15%)
	T0	0 (0.0%)	0 (0.0%)	0 (0.0%)	0 (0.0%)	0 (0.0%)		
	T1	171 (49.1%)	58 (28.0%)	111 (86.7%)	1 (33.3%)	1 (10.0%)		
	T2	20 (5.7%)	19 (9.2%)	0 (0.0%)	1 (33.3%)	0 (0.0%)		
	T3	157 (45.1%)	130 (62.8%)	17 (13.3%)	1 (33.3%)	9 (90.0%)		
Stage N	NX	305 (88.4%)	164 (82.0%)	124 (98.4%)	2 (66.7%)	15 (93.8%)	<0.001	63 (15%)
	N0	25 (7.2%)	23 (11.5%)	2 (1.6%)	0 (0.0%)	0 (0.0%)		
	N1	13 (3.8%)	12 (6.0%)	0 (0.0%)	1 (33.3%)	0 (0.0%)		
	N2	1 (0.3%)	0 (0.0%)	0 (0.0%)	0 (0.0%)	1 (6.3%)		
	N3	1 (0.3%)	1 (0.5%)	0 (0.0%)	0 (0.0%)	0 (0.0%)		
Stage M	MX	342 (97.2%)	195 (95.6%)	126 (100.0%)	3 (100.0%)	18 (94.7%)	0.133	56 (14%)
	M0	2 (0.6%)	2 (1.0%)	0 (0.0%)	0 (0.0%)	0 (0.0%)		
	M1	8 (2.3%)	7 (3.4%)	0 (0.0%)	0 (0.0%)	1 (5.3%)		
Started Procedure Type	Open	70 (17.2%)	38 (16.5%)	27 (17.4%)	2 (66.7%)	3 (15.8%)	<0.001	0 (0%)
	Laparoscopy	241 (59.1%)	187 (81.0%)	40 (25.8%)	1 (33.3%)	13 (68.4%)		
	Robotic	97 (23.8%)	6 (2.6%)	88 (56.8%)	0 (0.0%)	3 (15.8%)		
Side	Right	199 (49.1%)	110 (48.0%)	79 (51.3%)	1 (33.3%)	9 (47.4%)	0.294	3 (0.7%)
	Left	196 (48.4%)	112 (48.9%)	73 (47.4%)	1 (33.3%)	10 (52.6%)		
	Both	10 (2.5%)	7 (3.1%)	2 (1.3%)	1 (33.3%)	0 (0.0%)		
Converted to Open Surgery	Yes	16 (4.5%)	7 (3.4%)	8 (5.8%)	0 (0.0%)	1 (5.9%)	0.443	49 (12%)
Operation Time (min)	Minute	202.0 (161.0, 258.0)	180.0 (138.5, 226.0)	221.5 (180.0, 279.0)	232.0 (220.0, 243.5)	300.0 (290.0, 352.0)	<0.001	136 (33%)
Est. Blood Loss (mL)	mL	200.0						
(150.0, 500.0)	200.0							
(100.0, 500.0)	200.0							
(150.0, 400.0)	300.0							
(300.0, 650.0)	350.0							
(220.0, 500.0)	0.114	128 (31%)						
Blood Transfusion	Yes	25 (6.3%)	19 (8.5%)	5 (3.2%)	1 (33.3%)	0 (0.0%)	0.033	9 (2.2%)
Hospital Stay (days)	Days	5.0 (3.0, 8.0)	5.0 (3.0, 9.0)	4.0 (3.3, 7.0)	7.0 (5.0, 16.0)	11.0 (5.0, 16.5)	0.004	4 (1.0%)
Reasons of Conversion	Bleeding	6 (40.0%)	5 (71.4%)	1 (14.3%)	0 (NA%)	0 (0.0%)	0.069	393 (96%)
	Difficult dissection	3 (20.0%)	0 (0.0%)	2 (28.6%)	0 (NA%)	1 (100.0%)		
	Failure to progress	2 (13.3%)	0 (0.0%)	2 (28.6%)	0 (NA%)	0 (0.0%)		
	Obesity	1 (6.7%)	0 (0.0%)	1 (14.3%)	0 (NA%)	0 (0.0%)		
	Other	3 (20.0%)	2 (28.6%)	1 (14.3%)	0 (NA%)	0 (0.0%)		
Intraoperative Complications	Yes	17 (4.2%)	13 (5.6%)	2 (1.3%)	1 (33.3%)	1 (5.3%)	0.02	0 (0%)
Postoperative Complications	Yes	34 (8.3%)	21 (9.1%)	11 (7.1%)	2 (66.7%)	0 (0.0%)	0.025	0 (0%)
Clavien Classification	No complications	307 (82.1%)	168 (79.6%)	123 (87.2%)	0 (0.0%)	16 (84.2%)	0.003	34 (8.3%)
	1	15 (4.0%)	7 (3.3%)	6 (4.3%)	1 (33.3%)	1 (5.3%)		
	2	10 (2.7%)	6 (2.8%)	3 (2.1%)	0 (0.0%)	1 (5.3%)		
	3	14 (3.7%)	7 (3.3%)	6 (4.3%)	0 (0.0%)	1 (5.3%)		
	4	12 (3.2%)	9 (4.3%)	1 (0.7%)	2 (66.7%)	0 (0.0%)		
	None	16 (4.3%)	14 (6.6%)	2 (1.4%)	0 (0.0%)	0 (0.0%)		
Death	Yes	19 (4.7%)	11 (4.8%)	3 (1.9%)	1 (33.3%)	4 (21.1%)	0.002	0 (0%)
Local Recurrence	Yes	12 (3.1%)	9 (4.1%)	2 (1.4%)	1 (33.3%)	0 (0.0%)	0.06	20 (4.9%)
Metastatic Progression	Yes	44 (11.3%)	32 (14.5%)	4 (2.7%)	1 (33.3%)	7 (36.8%)	<0.001	20 (4.9%)
Chemotherapy	Yes	26 (6.7%)	18 (8.2%)	3 (2.0%)	1 (33.3%)	4 (21.1%)	0.001	18 (4.4%)

## Discussion

Open radical nephrectomy was once the standard of care for all renal tumors, but its indications are now decreasing in tandem with the increasing adoption of MIS nephrectomies, especially for stage 1 tumors (1a < 4 cm, 1b = 4–7 cm) (15, 16). Our results align with this trend, showing a significant difference in the mean tumor size between procedure types (12). Similarly, robotic nephrectomies (which were mostly partial) were mainly performed for stage T1 tumors, while most open and laparoscopic surgeries (which were mostly radical) were mainly performed for stage T3 tumors. MIS significantly reduced perioperative morbidity in cancer patients, including estimated blood loss, duration of hospital stay, intraoperative complications, and postoperative complications (17). In addition, we observed significant differences in renal function across procedure types with robotic surgery featuring a higher mean eGFR and lower serum creatinine and BUN values at 1-year post-surgery, but this does not indicate that the robotic technique is in itself associated with better preservation of renal function because most robotic surgeries were partial nephrectomies (90.5%), which are known to better preserve renal function, while only 20.7% of open and 13.7% of laparoscopic surgeries were partial nephrectomies. However, overall survival of patients across the different procedure approaches was not statistically significant (p = 0.054), which agrees with previous research demonstrating the equivalence of MIS and open nephrectomy in terms of overall survival (18, 19). Recent data show a significant survival advantage for patients who underwent MIS radical nephrectomy compared to open radical nephrectomy, but no significant difference is observed in locally advanced stage 3 disease (19, 20). The lack of a subgroup analysis distinguishing between the survival of early stage and late-stage cancer patients undergoing MIS and open nephrectomy in our cohort limits our ability to further clarify this discrepancy. Nevertheless, our results support the established data regarding the safety and effectiveness of MIS in renal cancer patients (19, 21, 22).

Most nephrectomies at our institute, radical or partial, were performed using MIS, with 81.0% of radical nephrectomies being laparoscopic and 56.8% of partial nephrectomies being robotic. This contrasts with the study by Asker et al. (14), where laparoscopic partial nephrectomies were more common than robotic (42.0% vs 31.9%, respectively), and the Alhaidari et al. study (12), where open radical nephrectomies were more common than robotic (60 vs 40%, respectively); hence, our findings reflect an increasing trend toward MIS for both radical and partial nephrectomies at tertiary care centers in Saudi Arabia. Nevertheless, radical nephrectomy is usually performed for patients with more complex tumors, which likely explain their higher incidence of blood transfusions, perioperative complications, longer hospital stays, and worse oncological and survival outcomes (12, 23, 24). When solely considering early stage tumors (T1 and T2), studies did not suggest a markedly improved survival associated with partial nephrectomy (25, 26), although it is still preferred due to a significantly lower risk of developing CKD and cardiovascular events post-surgery (25–27).

A major limitation of the overall analysis is that patients undergoing open nephrectomies had likely more complex tumors compared to the minimally invasive group, which can explain their worse surgical and oncological outcomes. Therefore, our group recently performed a subgroup analysis of outcomes of locally advanced stage T3 renal cancer patients (from the cohort of the present study) who underwent radical nephrectomy (mainly performed openly/laparoscopically) or partial nephrectomy (mainly performed robotically) to evaluate the use of MIS in more complex cases (28). T3 RCC, or locally advanced RCC, is defined as tumor extension beyond the renal capsule into the venous or collecting systems or invasion into the peripheric or renal sinus fat (29). Stage T3 RCC was historically considered an absolute indication for radical nephrectomy (RN), and this remains the standard per current guidelines (30). However, recent studies have challenged this notion and suggested a place for partial nephrectomy (PN )in the management of these lesions. An RCC stage of T3 and beyond imparts a significantly greater risk of preoperative CKD (31), making renal preservation an increasingly important consideration to improve long-term patient outcomes in terms of function and quality of life. Our results indicated that PN, predominantly performed robotically at our institute, achieves comparable oncological outcomes of local recurrence (p=0.597), metastatic progression (p=0.129), chemotherapy use (p=0.367), and survival outcomes (log-rank p-value = 0.852) based on the type of nephrectomy (28). Andrade et al. similarly demonstrated comparable 3-year cancer-specific survival and recurrence-free survival rates between robot-assisted PN and robot-assisted PN, but significantly better preservation of renal function in the latter (32). Other studies investigating the utility of PN in patients clinically upstaged to stage T3 have also demonstrated statistically insignificant differences in oncological outcomes between PN and RN, with the former offering superior renal preservation (33–35). A study by Yim et al. studying PN for stage T3 renal cancer reported that 64% of patients achieved a trifecta, of which 37.6% achieved an optimal outcome, defined as patients who additionally preserved > 90% of their eGFR and had no increase in their CKD stage (36). A multivariate analysis identified significant predictors of failure to achieve a trifecta or optimal outcome, including higher age, increased RENAL nephrometry score, and an intraoperative blood loss > 300 mL (36). Our results show a significantly younger age in stage T3 patients who underwent PN, which may partly explain the comparable outcomes achieved in the radical group. Overall, therefore, MIS approaches may offer good oncological outcomes even in more complex cases while offering better preservation of renal function and improving operative parameters.

## Limitations

The single-center nature and small sample size limit the generalizability of our findings. Retrospective studies also have inherent limitations such as missing data and inaccurate documentation, which may introduce information bias.

## Conclusion

The present study is the most comprehensive study on nephrectomy outcomes in Saudi Arabia. Our findings are consistent with published data on the safety and efficacy of MIS nephrectomies in terms of reducing inpatient morbidity and hospital stay while conferring equivalent oncological and surgical outcomes. We also highlighted current gaps in our knowledge—particularly the use of partial nephrectomies in later stage renal cancer—for future research to address.

## Data Availability

Not applicable

## References

[1] Scosyrev E, Messing EM, Sylvester R, Campbell S, Van Poppel H. Renal function after nephron-sparing surgery versus radical nephrectomy: Results from EORTC randomized trial 30904. European Urology. 2014;65(2):372–7. 10.1016/j.eururo.2013.06.044 23850254

[2] Campbell Steven C, Clark Peter E, Chang Sam S, Karam Jose A, Souter L, Uzzo Robert G. Renal mass and localized renal cancer: Evaluation, management, and follow-up: AUA Guideline: Part I. Journal of Urology. 2021;206(2):199–208. 10.1097/JU.0000000000001911 34115547

[3] Porpiglia F, Volpe A, Billia M, Scarpa RM. Laparoscopic versus open partial nephrectomy: Analysis of the current literature. European Urology. 2008;53(4):732–43. 10.1016/j.eururo.2008.01.025 18222599

[4] Nanidis TG, Antcliffe D, Kokkinos C, Borysiewicz CA, Darzi AW, Tekkis PP, et al. Laparoscopic versus open live donor nephrectomy in renal transplantation: A meta-analysis. Annals of Surgery. 2008;247(1):58–70. 10.1097/SLA.0b013e318153fd13 18156924

[5] Aboumarzouk OM, Stein RJ, Eyraud R, Haber G-P, Chlosta PL, Somani BK, et al. Robotic versus laparoscopic partial nephrectomy: A systematic review and meta-analysis. European Urology. 2012;62(6):1023–33. 10.1016/j.eururo.2012.06.038 22771266

[6] Wilson CH, Sanni A, Rix DA, Soomro NA. Laparoscopic versus open nephrectomy for live kidney donors. Cochrane Database of Systematic Reviews. 2011(11). 10.1002/14651858.CD006124.pub2 22071829

[7] Dunn Matthew D, Portis Andrew J, Shalhav Arieh L, Elbahnasy Abdelhamid M, Heidorn C, McDougall Elspeth M, et al. Laparoscopic versus open radical nephrectomy: A 9-year experience. Journal of Urology. 2000;164(4):1153–9. 10.1016/S0022-5347(05)67131-5 10992356

[8] Ruiz Guerrero E, Claro AVO, Ledo Cepero MJ, Soto Delgado M, Álvarez-Ossorio Fernández JL. Robotic versus laparoscopic partial nephrectomy in the new era: Systematic review. Cancers. 2023;15(6):1793. 10.3390/cancers15061793 36980679 PMC10046669

[9] Campbell SC, Novick AC, Belldegrun A, Blute ML, Chow GK, Derweesh IH, et al. Guideline for management of the clinical T1 renal mass. The Journal of Urology. 2009; 182(4):1271–9. 10.1016/j.juro.2009.07.004 19683266

[10] Mousa D, Alharbi A, Helal I, Al-Homrany M, Alhujaili F, Alhweish A, et al. Prevalence and associated factors of chronic kidney disease among relatives of hemodialysis patients in Saudi Arabia. Kidney International Reports. 2021;6(3):817–20. 10.1016/j.ekir.2020.12.029 33732996 PMC7938070

[11] Alkhateeb SS, Alothman AS, Addar AM, Alqahtani RA, Mansi TM, Masuadi EM. Kidney cancer in Saudi Arabia. A 25-year analysis of epidemiology and risk factors in a tertiary center. Saudi Medical Journal. 2018;39(5):459–63. 10.15537/smj.2018.5.22641 29738004 PMC6118183

[12] Alhaidari OI, Moazin MS, Kokandi AA, Alhussein RM, Alghaith AA. Robotic nephrectomy vs open nephrectomy: Comparison of complications and oncological outcomes. The Ulutas Medical Journal. 2018; 4:221–6. 10.5455/umj.20180913113022

[13] Seyam RM, Alalawi MM, Alkhudair WK, Alzahrani HM, Azhar RA, Alothman KI, et al. Operative outcomes of robotic partial nephrectomy. A report of the first 101 cases from a single center in Saudi Arabia. Saudi Medical Journal. 2019;40(1):33–40. 10.15537/smj.2019.1.22782 30617378 PMC6452606

[14] Asker AA, Addar A, Alghamdi M, Alawad S, Alharbi M, Hamri SB, et al. Partial nephrectomy, a comparison between different modalities: A tertiary care center experience. Journal of Kidney Cancer VHL. 2021;8(2):34–9. 10.15586/jkcvhl.v8i2.179 34178584 PMC8215000

[15] Thompson RH, Boorjian SA, Lohse CM, Leibovich BC, Kwon ED, Cheville JC, et al. Radical nephrectomy for pT1a renal masses may be associated with decreased overall survival compared with partial nephrectomy. Journal of Urology. 2008;179(2):468–71; Discussion 72–3. 10.1016/j.juro.2007.09.077 18076931

[16] Leibovich BC, Blute M, Cheville JC, Lohse CM, Weaver AL, Zincke H. Nephron sparing surgery for appropriately selected renal cell carcinoma between 4 and 7 cm results in outcome similar to radical nephrectomy. Journal of Urology. 2004;171(3):1066–70. 10.1097/01.ju.0000113274.40885.db 14767272

[17] Crocerossa F, Carbonara U, Cantiello F, Marchioni M, Ditonno P, Mir MC, et al. Robot-assisted radical nephrectomy: A systematic review and meta-analysis of comparative studies. European Urology. 2021;80(4):428–39. 10.1016/j.eururo.2020.10.034 33218826

[18] Auffenberg Gregory B, Curry M, Gennarelli R, Blum Kyle A, Elkin E, Russo P. Comparison of cancer-specific outcomes following minimally invasive and open surgical resection of early stage kidney cancer from a national cancer registry. Journal of Urology. 2020;203(6):1094–100. 10.1097/JU.0000000000000741 31913076 PMC8498972

[19] Bragayrac LAN, Abbotoy D, Attwood K, Darwiche F, Hoffmeyer J, Kauffman EC, et al. Outcomes of minimal invasive vs open radical nephrectomy for the treatment of locally advanced renal-cell carcinoma. Journal of Endourology. 2016;30(8):871–6. 10.1089/end.2016.0082 27203682

[20] Dursun F, Elshabrawy A, Wang H, Rodriguez R, Liss MA, Kaushik D, et al. Survival after minimally invasive vs. open radical nephrectomy for stage I and II renal cell carcinoma. International Journal of Clinical Oncology. 2022;27(6):1068–76. 10.1007/s10147-022-02153-5 35319076

[21] Luciani LG, Porpiglia F, Cai T, D’Elia C, Vattovani V, Giusti G, et al. Operative safety and oncologic outcome of laparoscopic radical nephrectomy for renal cell carcinoma >7 cm: A multicenter study of 222 patients. Urology. 2013;81(6):1239–45. 10.1016/j.urology.2012.12.065 23608667

[22] Becher E, Jericevic D, Huang WC. Minimally invasive surgery for patients with locally advanced and/or metastatic renal cell Carcinoma. Urologic Clinics of North America. 2020;47(3):389–97. 10.1016/j.ucl.2020.04.004 32600540

[23] Mir MC, Derweesh I, Porpiglia F, Zargar H, Mottrie A, Autorino R. Partial nephrectomy versus radical nephrectomy for clinical T1b and T2 renal tumors: A systematic review and meta-analysis of comparative studies. European Urology. 2017;71(4):606–17. 10.1016/j.eururo.2016.08.060 27614693

[24] Simmons MN, Weight CJ, Gill IS. Laparoscopic radical versus partial nephrectomy for tumors >4 cm: Intermediate-term oncologic and functional outcomes. Urology. 2009;73(5):1077–82. 10.1016/j.urology.2008.11.059 19394509

[25] Gershman B, Thompson RH, Boorjian SA, Lohse CM, Costello BA, Cheville JC, et al. Radical versus partial nephrectomy for cT1 renal cell carcinoma. European Urology. 2018;74(6):825–32. 10.1016/j.eururo.2018.08.028 30262341

[26] Forbes CM, Rendon RA, Finelli A, Kapoor A, Moore RB, Breau RH, et al. Disease progression and kidney function after partial vs. radical nephrectomy for T1 renal cancer. Urologic Oncology. 2016;34(11):486.e17–.e23. 10.1016/j.urolonc.2016.05.034 27423824

[27] Ochoa-Arvizo M, García-Campa M, Santos-Santillana KM, Klatte T, García-Chairez LR, González-Colmenero AD, et al. Renal functional and cardiovascular outcomes of partial nephrectomy versus radical nephrectomy for renal tumors: A systematic review and meta-analysis. Urologic Oncology: Seminars and Original Investigations. 2023;41(3):113–24. 10.1016/j.urolonc.2022.11.024 36642639

[28] Alasker A, Alnafisah TR, Alghafees M, Shafqat A, Sabbah BN, Alhaider A, et al. Preserving renal function without compromising oncological outcomes: A comparative study of partial and total nephrectomies in T3 stage renal cell carcinoma. Journal of Kidney Cancer and VHL. 2023;10(4):28–32. 10.15586/jkcvhl.v10i4.290 38162464 PMC10755761

[29] Warren AY, Harrison D. WHO/ISUP classification, grading and pathological staging of renal cell carcinoma: Standards and controversies. World Journal of Urology. 2018;36(12):1913–26. 10.1007/s00345-018-2447-8 30123932 PMC6280811

[30] Dhanji S, Wang L, Liu F, Meagher MF, Saidian A, Derweesh IH. Recent advances in the management of localized and locally advanced renal cell carcinoma: A narrative review. Research and Reports in Urology. 2023:99–108. 10.2147/RRU.S326987 36879830 PMC9985462

[31] Dey S, Hamilton Z, Noyes SL, Tobert CM, Keeley J, Derweesh IH, et al. Chronic kidney disease is more common in locally advanced renal cell carcinoma. Urology. 2017;105:101–7. 10.1016/j.urology.2017.03.033 28365357

[32] Hiury S. Andrade, Homayoun Zargar, Oktay Akca, Onder Kara, Peter A. Caputo, Daniel Ramirez, et al. Is robotic partial nephrectomy safe for T3a renal cell carcinoma? Experience of a high-volume center. Journal of Endourology. 2017;31(2):153–7. 10.1089/end.2016.0622 27881027

[33] Patel SH, Uzzo RG, Larcher A, Peyronnet B, Lane BR, Pruthi D, et al. Oncologic and functional outcomes of radical and partial nephrectomy in pT3a pathologically upstaged renal cell carcinoma: A multi-institutional analysis. Clinical Genitourinary Cancer. 2020;18(6):e723–e9. 10.1016/j.clgc.2020.05.002 32600941

[34] Shvero A, Nativ O, Abu-Ghanem Y, Zilberman D, Zaher B, Levitt M, et al. Oncologic outcomes of partial nephrectomy for stage T3a renal cell cancer. Clinical Genitourinary Cancer. 2018;16(3):e613–e7. 10.1016/j.clgc.2017.10.016 29174471

[35] Maurice MJ, Zhu H, Kim S, Abouassaly R. Survival after partial and radical nephrectomy for high-risk disease: A propensity-matched comparison. Canadian Urological Association Journal. 2016;10(9–10):e282–e9. 10.5489/cuaj.3707 27695581 PMC5028211

[36] Yim K, Aron M, Rha KH, Simone G, Minervini A, Challacombe B, et al. Outcomes of robot-assisted partial nephrectomy for clinical T3a renal masses: A multicenter analysis. European Urology Focus. 2021;7(5):1107–14. 10.1016/j.euf.2020.10.011 33249089

